# Mediation role of estimated pulse wave velocity in the association between physical activity and cognitive function

**DOI:** 10.1097/MD.0000000000048085

**Published:** 2026-04-03

**Authors:** Yaxiong Zheng, Hwi-Yeol Yun, Jae-Hyun Lee, Wooyeon Jo, Jaeho Jin, Soyoon Lee, Seyeon Jang, Sang Ki Lee

**Affiliations:** a Department of Sport Science, College of Natural Science, Chungnam National University, Daejeon, Yuseong-gu, Korea; b College of Pharmacy, Chungnam National University, Daejeon, Yuseong-gu, Korea.

**Keywords:** cognitive function, estimated pulse wave velocity, NHANES, physical activity

## Abstract

This study aimed to clarify the mediating role of arterial stiffness, measured as estimated pulse wave velocity (ePWV), in the relationship between physical activity levels and cognitive function in older adults. Data from the National Health and Nutrition Examination Survey from 2011 to 2014 were analyzed. ePWV was calculated using age and mean blood pressure. Survey-weighted logistic regression and subgroup analyses were conducted to explore the associations between physical activity and cognitive function. Additionally, receiver operating characteristic curve analysis assessed the predictive value of ePWV for cognitive impairment. Mediation analysis was used to determine whether ePWV mediates the relationship between physical activity and cognitive function. A total of 1802 participants aged 60 years or older were included in the study. Higher physical activity levels were significantly associated with better cognitive function after adjusting for confounders (OR: 1.517, 95% CI: 1.010–2.280, *P* = .046). Subgroup analyses confirmed a consistent positive association across most covariates. Receiver operating characteristic curve analysis showed that ePWV had moderate predictive value for cognitive impairment (area under the curve: 0.678, 95% CI: 0.647–0.709). Mediation analysis revealed that ePWV partially mediated the relationship between physical activity and cognitive impairment, with a total effect of 0.031, a mediated effect of 0.006, and a proportion mediated of 19.35%. Higher physical activity levels are associated with better cognitive function in older adults. Estimated pulse wave velocity plays a mediating role in this association, suggesting that arterial stiffness may be an important pathway through which physical activity benefits cognitive health.

## 1. Introduction

Cognitive functions encompass crucial aspects such as memory, language, executive functions, attention, and reaction abilities,^[1]^ which are fundamental in sustaining our daily life quality. Aging is often accompanied by a natural decline in cognitive function, posing a significant challenge, particularly for older adults. The impact of cognitive decline on older adults is substantial, leading to a heightened risk of diminished quality of life and increased limitations in daily activities.^[2]^ In the United States alone, a staggering 6.7 million individuals aged 65 years or more are grappling with Alzheimer’s disease, a consequence of cognitive decline.^[3]^ This imposes substantial economic and societal burdens.^[4]^ This number is projected to surge to 13.8 million by 2060.^[5]^ Concurrently, mounting evidence has linked declining cognitive function in the elderly to an elevated risk of mortality.^[6,7]^ Consequently, prioritizing research efforts to prevent and decelerate cognitive decline becomes paramount for the well-being of aging populations.

Numerous epidemiological studies have demonstrated that cognitive decline is correlated with various lifestyle factors, including sleep patterns, living environment, smoking, diet, social interaction, and physical activity.^[8–13]^ Notably, physical activity has emerged as a crucial factor in preventing and decelerating cognitive decline.^[14–16]^ Its positive impact stems from mechanisms such as elevating growth factor levels, inhibiting tau phosphorylation, reducing amyloid β concentration, regulating inflammatory cytokines, enhancing cerebral blood flow, and alleviating oxidative stress.^[17]^ Several recent studies have shown that physical activity can improve cognitive function by reducing vascular risk factors.^[18]^ There is a mounting body of evidence highlighting the adverse impact of vascular risk factors on cognitive function.^[19]^ Arterial blood vessel stiffness, a key indicator of vascular health, is commonly assessed through pulse wave velocity, which reflects the speed of pulse wave propagation through arterial blood vessels.^[20]^ Among various methods for evaluating arterial stiffness, carotid-femoral pulse wave velocity (cfPWV) is the most recognized and established index.^[21]^ However, the application of cfPWV in clinical settings is constrained by specialized equipment and expertise. In response, researchers have developed an equation known as estimated pulse wave velocity (ePWV) that can be calculated based on age and blood pressure. This estimation offers a practical means to assess arterial stiffness. Multiple studies have confirmed that ePWV and cfPWV share comparable predictive value in measuring vascular aging and forecasting cardiovascular risk factors.^[22,23]^ Based on such background, the present study aimed to investigate the relationship between physical activity and cognitive function, concurrently exploring the mediating role of ePWV in the association between physical activity and cognitive function utilizing data from the National Health and Nutrition Examination Survey.

## 2. Methods

### 2.1. Data collection and study population

The National Health and Nutrition Examination Survey (NHANES) database has been conducting nationally representative cross-sectional surveys of the civilian noninstitutional population since 1999 to assess nutritional and health status of the U.S. population (Centers for Disease Control and Prevention). Data were collected in a 2-year cycle. Each cycle follows a complex stratified multistage probability sampling design.^[24]^ The survey comprises a home interview conducted in the respondent’s home, followed by physical and laboratory examinations at a specially designed mobile examination center. Ethical approval for all study protocols in NHANES was granted by the National Center for Health Statistics Research Ethics Review Board. Written informed consent was obtained from all participants before the study. Since the database is publicly available, no external ethics approval or administrative permission is required. All analytical procedures in this study were carried out in strict accordance with NHANES guidelines and regulations.

**Figure 1.**
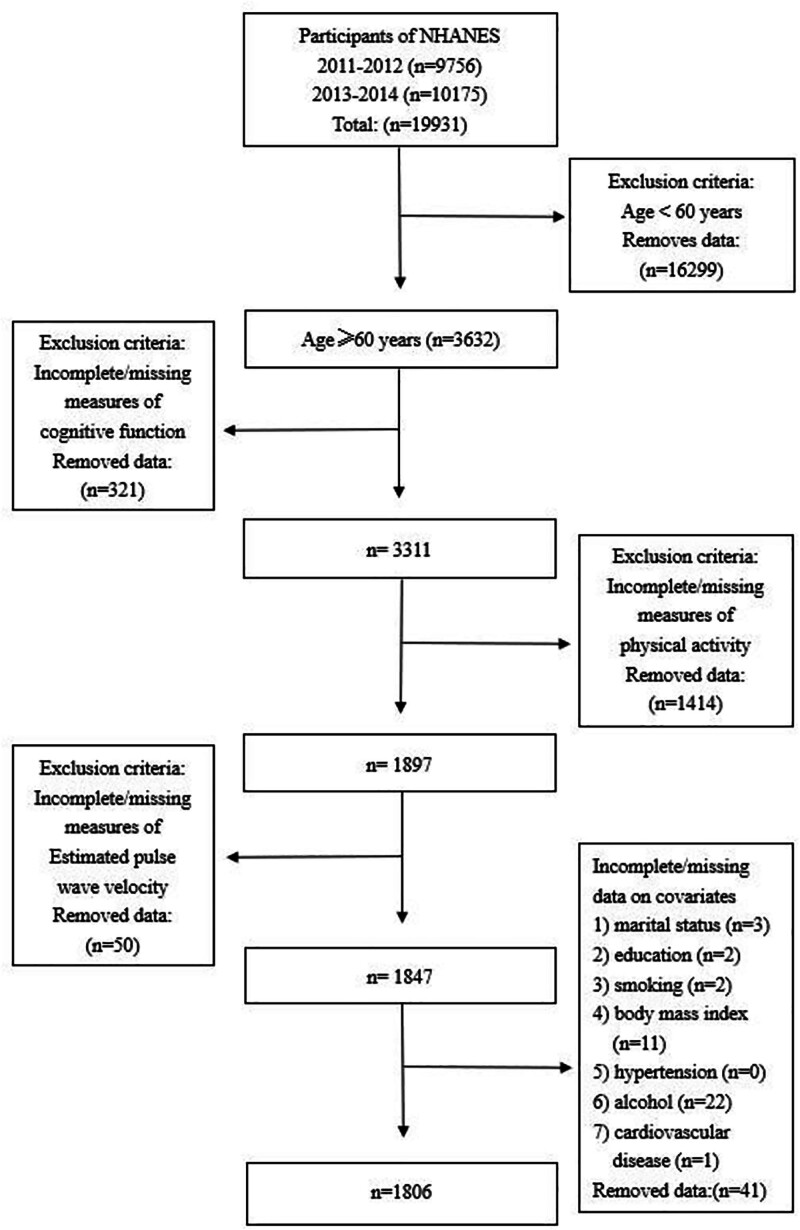
Flow chart showing the selection of study subjects with inclusion and exclusion criteria.

A thorough search of the NHANES database revealed that cognitive function tests were exclusively conducted for individuals aged ≥60 years, limited to the 2 cycles of 2011 to 2012 and 2013 to 2014. Consequently, this study included elderly participants aged ≥60 years during a 4-year period from 2011 to 2014. Participants were eligible if they were aged ≥60 years and had complete data for physical activity, cognitive function, blood pressure, and covariates. We excluded those with missing or extreme values based on NHANES guidelines. The final analytical sample consisted of 1806 participants (original n = 3632; excluded due to missing data = 1826). The screening process flowchart is illustrated in Figure 1.

### 2.2. Physical activity

Physical activity was assessed using the Global Physical Activity Questionnaire included in NHANES. We used data from the Physical Activity Questionnaire files (PAQ_H for 2011–2012 and PAQ_I for 2013–2014). Specifically, vigorous work activity, moderate work activity, recreational activity, and transportation activity such as walking or bicycling were extracted. Weekly metabolic equivalent (MET)-minutes were calculated following World Health Organization and NHANES analytical guidelines. For each activity, the formula was: MET-min/week = days per week × minutes per day × assigned MET value. We assigned 8 METs for vigorous activity, 4 METs for moderate activity, and 4 METs for walking/bicycling. Total weekly MET-minutes were obtained by summing across all activity domains. Following the Physical Activity Guidelines for Americans, individuals were considered adequately active if they achieved at least 150 minutes of moderate-intensity physical activity per week, corresponding to a minimum MET score of 600.^[25]^ The World Health Organization identified those with MET scores below 600 as insufficiently active.^[26]^

### 2.3. Estimated pulse wave velocity

ePWV was calculated from the equation of age and mean blood pressure (MBP). It was expressed in m/s.^[22]^ ePWV was calculated with the following formula:ePWV = 9.587 − (0.402 × age) + (4.560 × 10^-3^ × age^2^) ‐ (2.621 × 10^-5^ × age^2^ × MBP) + (3.176 × 10^-3^ × age × MBP) − (1.832 × 10^-2^ × MBP).^[27]^ In this formula, age was given in years and MBP was calculated as DBP + 0.4 (SBP − DBP), where DBP was diastolic blood pressure and SBP was systolic blood pressure. Both were obtained by trained NHANES technicians. Participants took 3 consecutive blood pressure measurements after sitting quietly for 5 minutes. The average of these 3 blood pressure measurements was used to determine diastolic blood pressure and systolic blood pressure.

### 2.4. Cognitive function

Cognitive function was assessed by trained technicians at the mobile examination center for participants aged 60 years or older. The validity and reliability of these instruments in older U.S. populations have been well established. Specifically, the Consortium to Establish a Registry for Alzheimer’s Disease (CERAD) Word Learning Test is a reliable tool for detecting early cognitive decline, the animal fluency test (AFT) is widely applied to measure executive functioning in epidemiological studies of aging, and the digit symbol substitution test (DSST) is highly sensitive to age-related decline in processing speed and predictive of dementia onset. Moreover, previous NHANES-based studies have consistently confirmed that these tests are appropriate and effective for evaluating cognitive function in community-dwelling older adults.^[28–30]^

*CERAD test*: This test comprised 3 consecutive immediate recall sessions and a delayed recall test. Participants verbally recited 10 unrelated words displayed on a computer monitor and subsequently recalled them immediately after reading. Three immediate recall tests were conducted, each with a different order of the 10 words. Following completion of the AFT and DSST, participants underwent a delayed recall test. Each correct answer was assigned one point. CERAD scores ranged from 0 to 40 points, with a higher score indicating a better cognitive ability.^[31]^ AFT was used to assess verbal fluency and processing speed. Participants were tasked with naming as many animals as possible within 1 minute, with each named animal earning one point.^[32]^ DSST was used to evaluate sustained attention and executive function. Administered on a single sheet of paper, subjects matched symbols to numbers based on a key provided at the top of the page. Correct copying of symbols within a specified time (usually 90–120 s) was used to determine the performance.^[29]^

To provide a comprehensive evaluation of overall cognitive function, scores from CERAD, AFT, and DSST tests were combined, constituting the global cognitive function (GCF).^[33]^ In the absence of clear criteria for individual test judgments, quartile statistical analysis employed the lowest quartile (25th percentile) as the cutoff point for cognitive impairment. Participants scoring within this range were categorized into a cognitive impairment group, while those exceeding this threshold were classified as having noncognitive impairment.^[34]^ Cutoff points of CERAD test, AFT, DSST, and GCF were 21, 13, 36, and 73, respectively.

### 2.5. Covariates

Multivariable logistic regression models were constructed to control for potential confounders. Covariates were selected a priori based on previous literature and clinical relevance to both physical activity and cognitive function, as well as their potential associations with vascular aging. These included demographic characteristics (gender, age, ethnicity, marital status, and education), lifestyle factors (cigarette smoking and alcohol use), and clinical conditions (body mass index [BMI], hypertension, and cardiovascular disease). All covariates were simultaneously entered into the models to adjust for residual confounding, and NHANES sampling weights, strata, and primary sampling units were applied to account for the complex survey design and to ensure robustness of the findings.

Demographic characteristics were classified as follows: gender (male and female); age (60–<70 years, 70–<80 years, and ≥80 years) considering the impact of age on analysis results^[35]^; ethnicity (Mexican American, non-Hispanic Black, non-Hispanic White, other Hispanic, and other race including multiracial); marital status (never married, living with partner, married, divorced, separated, and widowed); and education (<9th grade, 9–11th grade includes 12th grade with no diploma, high school graduate/GED or equivalent, college, and college graduate or above). Lifestyle characteristics were categorized as follows: smoking status (never, <100 cigarettes during their lifetime; past smoker, ≥100 cigarettes during their lifetime and had quit smoking before the time of the study; or current smoker, ≥100 cigarettes during their lifetime and was smoking at the time of the study)^[36]^; drinking status (never drinking, <12 drinks in life; former drinking, ≥12 drinks in 1 year and did not drink last year; mild drinking, ≤1 drink per day in females and ≤2 drinks per day in males on average over the past 12 months; moderate drinking, 1 to 3 drinks per day for females and 2 to 4 drinks per day for males on average over the past 12 months; and heavy drinking, ≥4 drinks per day for women or ≥5 drinks per day for men on average over the past 12 months).^[37]^ Health status characteristics such as BMI = weight (kg) divided by height (m) squared,^[38]^ ever told to have a cardiovascular disease (CVD) or drugs for CVD use (yes, no),^[39]^ and hypertension (a systolic blood pressure ≥140 mm Hg, a diastolic blood pressure ≥90 mm Hg, or current use of antihypertensive medication [yes, no])^[40]^ were also evaluated.

### 2.6. Statistical analysis

All analyses followed NHANES analytic guidelines, accounting for complex sampling design using examination weights (wtmec2yr). Since 2 survey cycles (2011–2012 and 2013–2014) were combined, 4-year weights were created by dividing wtmec2yr by 2.^[41]^ To compare participants’ baseline characteristics, Student *t* test was employed for continuous variables and Chi-square test was used for categorical variables. Weighted multivariate logistic regression models were then applied to assess the relationship between physical activity and cognitive function. Subsequently, the statistical *P*-value for interactions between covariates and the physical activity level was calculated. Subgroup analyses were conducted to further clarify these results. To gauge the predictive efficacy of ePWV for cognitive impairment, a receiver operating characteristic (ROC) curve was used. The potential mediating effect of ePWV on the association between physical activity and cognitive function was estimated through parallel mediator analysis. All statistical analyses were executed using R Studio (version 4.3.1; Posit PBC, Boston) and IBM SPSS Statistics 27 (IBM Corp., Armonk). Statistical significance was established at *P* <.05.

## 3. Results

### 3.1. Population characteristics

Following stringent screening criteria, our study incorporated a total of 1806 participants, with 52.5% being males. Table 1 provides weighted baseline characteristics of study variables. The majority of participants fell within the 60 to 69 age group (58.4%). Non-Hispanic whites constituted the largest ethnic group (43.6%). Additionally, 76.0% of participants were married. Individuals with a college degree or higher education level accounted for 52.3%. The study population comprised 48.7% nonsmokers and 38.0% light drinkers. The prevalence of hypertension was notably high at 67.7%. Regarding overall cognitive function, a gender discrepancy was observed, with men more likely to exhibit lower cognitive function. Furthermore, individuals aged 70 to 79, non-Hispanic Black participants, and those with lower education levels demonstrated poorer cognitive functions. Noteworthy, participants with noncognitive impairment reported significantly higher weekly physical activity levels than those with cognitive impairment.

**Table 1:** Characteristics of participants in the NHANES 2011 to 2014 (N = 1806).

	CEARD test			Animal fluency test			Digit symbol substitution test			Global cognitive function		
Variables	Noncognitive impairment (n = 1328)	Cognitive impairment (n = 478)	*P*-value	Noncognitive impairment (n = 1350)	Cognitive impairment (n = 456)	*P*-value	Noncognitive impairment (n = 1354)	Cognitive impairment (n = 452)	*P*-value	Noncognitive impairment (n = 1349)	Cognitive impairment (n = 457)	*P*-value
Physical activity (MET-min/week)	2625.83 (110.57)	2172.06 (147.46)	.034	2606.43 (98.14)	2184.59 (183.54)	.049	2601.15 (90.62)	2092.58 (146.70)	.002	2622.45 (94.04)	1982.16 (177.54)	.002
ePWV (m/s)	12.21 (0.07)	13.10 (0.12)	<.001	12.30 (0.07)	12.81 (0.10)	<.001	12.28 (0.06)	13.11 (0.12)	<.001	12.25 (0.06)	13.27 (0.13)	<.001
BMI (kg/m^2^)	28.55 (0.21)	27.61 (0.31)	.006	28.46 (0.23)	27.86 (0.37)	.193	28.37 (0.22)	28.35 (0.28)	.968	28.43 (0.23)	27.97 (0.35)	.332
**Age: n (%**)			<.001			<.001			<.001			<.001
60–69 yr	844 (65.58)	205 (35.62)		814 (63.34)	235 (41.38)		822 (62.78)	227 (38.57)		840 (63.81)	209 (33.22)	
70–79 yr	360 (26.68)	159 (38.55)		374 (27.08)	145 (38.77)		380 (28.12)	139 (35.00)		371 (27.76)	148 (37.02)	
≥80 yr	124 (7.74)	114 (25.84)		162 (9.58)	76 (19.85)		152 (9.09)	86 (26.43)		138 (8.43)	100 (29.76)	
**Gender: n (%**)			<.001			.765			.405			.035
Female	695 (54.40)	163 (38.85)		642 (51.55)	216 (50.48)		681 (51.76)	177 (48.73)		681 (52.34)	177 (45.08)	
Male	633 (45.60)	315 (61.15)		708 (48.45)	240 (49.52)		673 (48.24)	275 (51.27)		668 (47.66)	280 (54.93)	
**Ethnicity: n (%**)			<.001			<.001			<.001			<.001
Mexican American	102 (2.67)	47 (4.65)		115 (2.80)	34 (4.38)		94 (2.23)	55 (8.79)		97 (2.30)	52 (8.01)	
Non-Hispanic black	302 (7.13)	112 (10.01)		256 (5.678)	158 (18.00)		263 (5.69)	151 (21.69)		270 (5.94)	144 (19.13)	
Non-Hispanic white	658 (82.19)	197 (71.88)		727 (84.28)	128 (59.15)		747 (84.80)	108 (47.82)		730 (84.41)	125 (52.47)	
Other Hispanic	119 (2.80)	75 (7.22)		134 (3.01)	60 (7.02)		90 (1.92)	104 (15.91)		94 (2.05)	100 (14.22)	
Other race	147 (5.22)	47 (6.24)		118 (4.24)	76 (11.46)		160 (5.36)	34 (5.80)		158 (5.30)	36 (6.16)	
**Marital status: n (%**)			.045			.008			<.001			<.001
Never married	72 (4.03)	28 (3.50)		76 (3.83)	24 (4.45)		71 (3.74)	29 (5.256)		73 (3.83)	27 (4.60)	
Living with partner	40 (2.62)	16 (2.16)		46 (2.64)	10 (1.96)		43 (2.48)	13 (2.87)		43 (2.59)	13 (2.11)	
Married	769 (65.27)	265 (61.55)		779 (65.36)	255 (60.38)		813 (66.90)	221 (48.07)		810 (66.46)	224 (52.03)	
Divorced	212 (14.00)	55 (11.63)		204 (14.01)	63 (11.13)		204 (13.55)	63 (13.45)		209 (13.95)	58 (10.85)	
Separated	31 (0.83)	18 (1.65)		29 (0.72)	20 (2.39)		19 (0.45)	30 (4.79)		21 (0.56)	28 (3.77)	
Widowed	204 (13.25)	96 (19.52)		216 (13.45)	84 (19.69)		204 (12.89)	96 (25.56)		193 (12.61)	107 (26.63)	
**Education: n (%**)			<.001			<.001			<.001			<.001
Less than 9th grade	67 (1.96)	107 (12.65)		96 (2.71)	78 (10.85)		26 (1.07)	148 (24.9)		31 (1.17)	143 (22.87)	
9–11th grade	146 (7.09)	78 (14.95)		140 (6.86)	84 (17.65)		131 (6.81)	93 (21.29)		135 (6.97)	89 (19.42)	
High school graduate	288 (18.97)	106 (25.66)		269 (18.52)	125 (29.26)		293 (19.59)	101 (25.00)		289 (19.42)	105 (25.83)	
College	443 (34.95)	107 (25.92)		444 (34.57)	106 (26.15)		479 (35.56)	71 (16.60)		473 (35.25)	77 (19.71)	
College graduate or above	384 (37.03)	80 (20.82)		401 (37.34)	63 (16.10)		425 (36.96)	39 (12.26)		421 (37.19)	43 (12.18)	
**Smoking status: n (%**)			.785			.115			.021			.191
Never	659 (49.04)	232 (50.85)		665 (49.34)	226 (49.67)		676 (49.56)	215 (48.25)		673 (49.58)	218 (48.17)	
Past smoker	515 (41.13)	182 (39.45)		533 (41.54)	164 (37.01)		537 (41.35)	160 (36.98)		530 (41.08)	167 (38.97)	
Current smoker	154 (9.83)	64 (9.70)		152 (9.12)	66 (13.32)		141 (9.10)	77 (14.77)		146 (9.34)	72 (12.86)	
**Alcohol use status: n (%**)			.007			<.001			<.001			<.001
Never	188 (10.96)	84 (16.15)		182 (10.95)	90 (17.21)		177 (10.72)	95 (20.71)		177 (10.78)	95 (19.75)	
Former	326 (19.59)	145 (25.70)		327 (19.25)	144 (28.59)		303 (18.09)	168 (39.60)		303 (18.14)	168 (38.05)	
Mild	570 (49.39)	178 (44.95)		590 (49.93)	158 (41.29)		640 (51.81)	108 (25.48)		630 (51.60)	118 (28.35)	
Moderate	153 (14.08)	35 (7.53)		154 (14.21)	34 (5.62)		150 (13.74)	38 (6.25)		151 (13.74)	37 (6.68)	
Heavy	91 (5.98)	36 (5.68)		97 (5.66)	30 (7.29)		84 (5.63)	43 (7.96)		88 (5.74)	39 (7.17)	
**CVD: n (%**)			.005			.454			.002			.006
No	1105 (83.2)	364 (74.17)		1108 (81.8)	361 (79.11)		1125 (82.6)	344 (73.26)		1119 (82.5)	350 (74.13)	
Yes	223 (16.82)	114 (25.83)		242 (18.11)	95 (20.89)		229 (17.40)	108 (26.74)		230 (17.45)	107 (25.87)	
**Hypertension: n (%**)			.016			.018			.002			<.001
No	446 (39.36)	145 (29.13)		459 (38.63)	132 (30.88)		471 (39.19)	120 (24.62)		471 (39.26)	120 (24.94)	
Yes	882 (60.65)	333 (70.87)		891 (61.37)	324 (69.12)		883 (60.82)	332 (75.38)		878 (60.74)	337 (75.06)	

Continuous variables are expressed as means (standard errors) and categorical variables are described as quantities (percentages). Continuous variables were compared in 2 groups using *t* test and categorical variables were compared using chi-square tests. *P* < .05 was set as the threshold of statistical significance.

BMI = body mass index, CVD = cardiovascular disease, ePWV = estimated pulse wave velocity, MET = metabolic equivalent, NHANES = National Health and Nutrition Examination Survey.

### 3.2. Weighted logistic regression analysis

To independently explore the relationship between physical activity and cognitive functions, we conducted weighted multivariate logistic regression analysis (Table 2). Table 2 presents fully adjusted models accounting for age, gender, ethnicity, marital status, education, alcohol use status, smoking status, BMI, CVD, and hypertension. The analysis encompassing the crude model (OR: 1.808, 95% CI: 1.283–2.546, *P* = .001), model 1 (OR: 1.612, 95% CI: 1.144–2.272, *P* = .008), model 2 (OR: 1.517, 95% CI: 1.061–2.171, *P* = .025), and model 3 (OR: 1.517, 95% CI: 1.010–2.280, *P* = .046), consistently supported a significant association between physical activity and cognitive impairment performance. These findings underscore the robustness of this relationship even after adjusting for various demographic and health-related variables.

**Table 2:** Weighted multivariate logistic regression to determine odds of cognitive impairment by physical activity level.

Global cognitive function	Crude model		Model 1		Model 2		Model 3	
Character	OR (95% CI)	*P*-value	OR (95% CI)	*P*-value	OR (95% CI)	*P*-value	OR (95% CI)	*P*-value
Normal physical activity	Reference		Reference		Reference		Reference	
Low physical activity	1.808 (1.283–2.546)	.001	1.612 (1.144–2.272)	.008	1.517 (1.061–2.171)	.025	1.517 (1.010–2.280)	.046

Crude model: not adjusted. Model 1: adjusted for age, gender, and ethnicity. Model 2: adjusted for age, gender, ethnicity, marital status, and education. Model 3: adjusted for age, gender, ethnicity, marital status, education, alcohol use status, smoking status, BMI, CVD, and hypertension.

95% CI = 95% confidence intervals, BMI = body mass index, CVD = cardiovascular disease, OR = odds ratio.

### 3.3. Subgroup analysis

To evaluate effects of covariates on the association between physical activity and cognitive function, participants were stratified based on age, gender, race, marital status, education, drinking status, smoking status, cardiovascular disease, and hypertension (Table 3). All subgroups showed no significant interactions with the association between physical activity and cognitive function (*P* for interaction > .05). ORs in all subgroups were greater than 1, indicating a robust positive relationship between physical activity and cognitive function. Although ORs were <1 in subgroups with separated of marital status (OR: 0.614, 95% CI: 0.049–7.754, *P = *.621), <9th grade of education (OR: 0.722, 95% CI: 0.259–2.013, *P* = .516), and heavy alcohol use status (OR: 0.809, 95% CI: 0.195–3.350, *P* = .761), *P*-values were not significant. These outcomes aligned with the earlier findings, emphasizing the robustness of the association across diverse demographic and health-related subgroups.

**Table 3:** Subgroup analyses on the association between physical activity level and cognitive function.

Character	OR (95% CI)	*P*-value	*P* for interaction
**Age**			.711
60–69 yr	1.881 (1.251–2.828)	.004	
70–79 yr	1.395 (0.795–2.449)	.237	
≥80 yr	1.679 (0.746–3.783)	.202	
**Gende**r			.848
Female	1.823 (1.160–2.864)	.011	
Male	1.938 (1.173–3.204)	.012	
**Ethnicity**			.514
Mexican American	1.252 (0.651–2.408)	.466	
Non-Hispanic black	1.219 (0.658–2.257)	.514	
Non-Hispanic white	1.970 (1.203–3.228)	.009	
Other Hispanic	1.950 (0.705–5.394)	.183	
Other race	1.333 (0.559–3.178)	.497	
**Marital status**			.703
Never married	3.111 (0.867–11.166)	.078	
Living with partner	1.985 (0.297–13.281)	.430	
Married	1.583 (0.989–2.534)	.055	
Divorced	2.020 (0.942–4.334)	.070	
Separated	0.614 (0.049–7.754)	.621	
Widowed	1.946 (1.032–3.671)	.040	
**Education**			.219
Less than 9th grade	0.722 (0.259–2.013)	.516	
9–11th grade	1.523 (0.596–3.887)	.364	
High school graduate	1.857 (1.047–3.295)	.035	
College	1.323 (0.757–2.312)	.313	
College graduate or above	3.158 (1.259–7.922)	.016	
**Smoking status**			.893
Never	1.924 (1.166–3.176)	.012	
Past smoker	1.659 (1.030–2.673)	.038	
Current smoker	1.775 (0.738–4.271)	.191	
**Alcohol use status**			.468
Never	2.143 (0.814–5.645)	.118	
Former	1.571 (0.948–2.605)	.078	
Mild	2.253 (1.379–3.683)	.002	
Moderate	1.229 (0.507–2.980)	.636	
Heavy	0.809 (0.195–3.350)	.761	
**CVD**			.717
No	1.952 (0.863–4.418)	.105	
Yes	1.687 (1.239–2.296)	.002	
**Hypertension**			.108
No	1.571 (1.091–2.261)	.017	
Yes	2.643 (1.428–4.894)	.003	

Model was adjusted for age, gender, ethnicity, marital status, education, smoking, alcohol use status, CVD, and hypertension. *P*-value was calculated by *P* for interaction and logistic regression analysis.

CVD = cardiovascular disease.

### 3.4. ROC analysis curves

**Figure 2.**
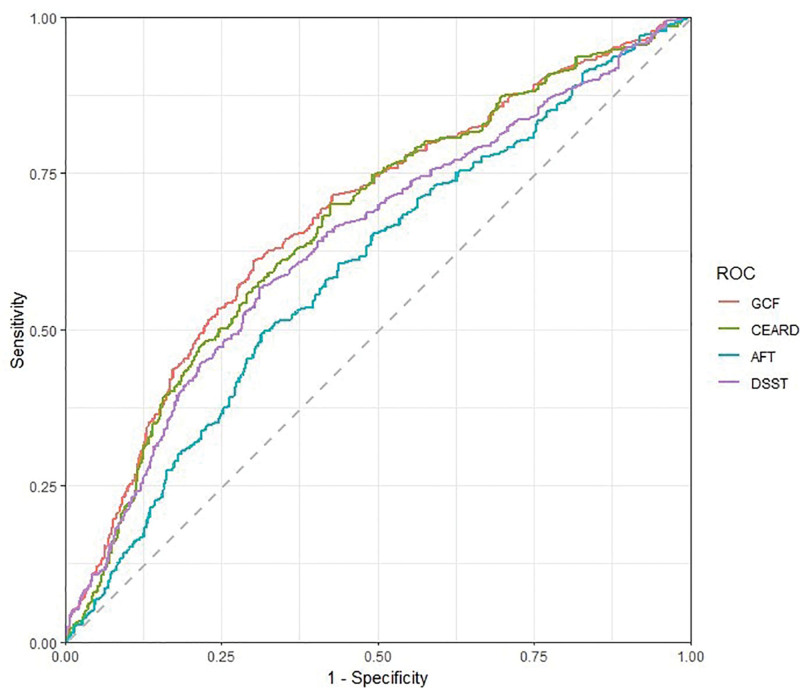
Receiver operator characteristic curve for ePWV to predict cognitive impairment. AFT = animal fluency test, CEARD = consortium to establish a registry for Alzheimer’s disease test, DSST = digit symbol substitution test, ePWV = estimated pulse wave, GCF = global cognitive function, ROC = receiver operator characteristic analysis curves.

ROC curves were generated for ePWV and cognitive impairment to assess the predictive capacity of ePWV for cognitive impairment, as illustrated in Figure 2. Area under the curve values for GCF, CEARD, AFT, and DSST were 0.678 (95% CI: 0.647–0.709), 0.666 (95% CI: 0.636–0.696), 0.595 (95% CI: 0.564–0.627), and 0.641 (95% CI: 0.610–0.672), respectively. These outcomes show that ePWV exhibits a partial predictive value for cognitive impairment.

### 3.5. Mediation analysis

**Figure 3.**
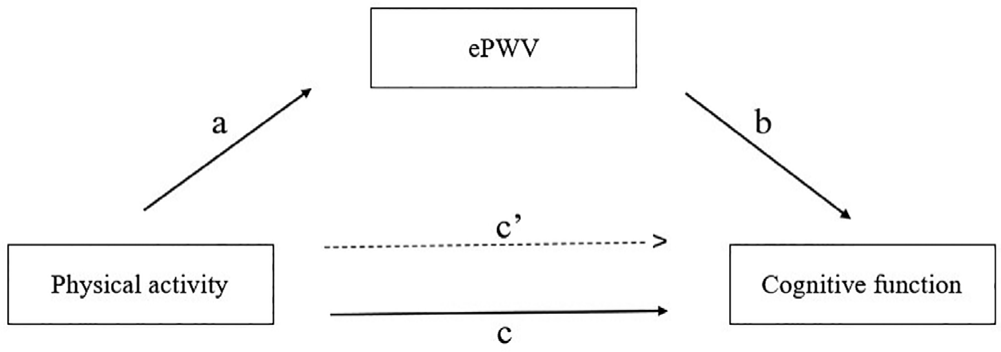
Path diagram of mediation analysis models. Path *a* indicates the regression coefficient for the association of physical activity with ePWV. Path *b* indicates the regression coefficient for the association of ePWV with cognitive function. Path *c* indicates the simple total effect of physical activity with cognitive function, without the adjustment for ePWV. Path *c*′ indicates the direct effect of physical activity with cognitive function when controlled for ePWV. ePWV = estimated pulse wave; *a* = *a* path; *b* = *b* path; *c* = *c* path; *c*′ = *c*′ path.

The mediation analysis aimed to discern whether and to what extent ePWV mediated the relationship between physical activity and cognitive function, employing 3 paths (a, b, and c) for assessment (Fig. 3). Path a gauged the connection between physical activity and ePWV (mediator). Pathway b evaluated the association between ePWV (mediator) and cognitive function. Path c represented the total effect of physical activity on cognitive function through ePWV, which was algebraically equivalent to *c* = *a* × *b* + *c′*. Path *c′* was the direct effect, reflecting the influence of physical activity on cognitive function controlling for ePWV. Mediation analysis results presented in Table 4 showed the following values: total effect = 0.031, direct effect = 0.025, mediated effect = 0.006, and proportion mediated = 19.35%. These results indicate that ePWV mediates the association between physical activity and cognitive function.

**Table 4:** Mediation effect of ePWV on the association between physical activity and cognitive function.

*c* total effect	*a* path	*a (P*-value)	*b* path	*b (P*-value)	*a* × *b* mediated effect	*a* × *b* (boot SE)	*a* × *b (Z* value)	*a* × *b (P*-value)	*a* × *b* (95% boot CI)	*c′* direct effect	Proportion mediated (%)
0.031	‐0.125	<.001	-0.046	<.001	0.006	0.002	3.679	<.001	0.009–0.003	0.025	19.35

*c* total effect = *a* path × *b* path + *c*′ direct effect. Mediated effect = *a* path × *b* path. Proportion mediated = mediated effect/*c* total effect. *P* < .05 was set as the threshold of statistical significance.

ePWV = estimated pulse wave velocity.

Sensitivity analyses confirmed the robustness of these findings. Mediation models with alternative covariate adjustments (age and sex only; main covariates; extended model including diabetes, cholesterol, and additional comorbidities), exclusion of participants with cardiovascular disease, trimming of extreme ePWV values (1st–99th percentile), and subgroup analyses stratified by sex and age groups all yielded indirect effects of approximately 0.005 to 0.006. Across these analyses, 95% bootstrap CIs did not cross zero (e.g., 0.003–0.009 in the main model), and the proportion mediated ranged from 15% to 22% (19.35% in the main model). Notably, small differences may occur due to rounding (e.g., 0.00575 displayed as 0.006), but the results consistently indicate stable partial mediation by ePWV (Table S1, Supplemental Digital Content, https://links.lww.com/MD/R594).

## 4. Discussion

It is well known that physical activity has a positive effect on cognitive function in older adults. To investigate the role of ePWV in the association between physical activity and cognitive function, we conducted a cross-sectional study involving 1806 U.S. adults aged ≥60 years. After adjusting for relevant covariates, we identified a significant positive association between physical activity and cognitive function. Insufficient weekly MET levels were linked to cognitive decline. Furthermore, ROC curve results indicated that ePWV also demonstrated a predictive value for cognitive impairment. Concurrently, our analysis revealed that physical activity could exert both a direct effect and an ePWV-mediated indirect effect on cognitive function.

Our findings align with a substantial body of evidence supporting a connection between physical activity and cognitive function. A positive correlation between physical activity and cognitive function has been consistently observed in the elderly.^[42–44]^ Increasing levels of physical activity have been shown to enhance various cognitive functions such as animal verbal fluency, immediate and delayed recall, as well as Digit Symbol Substitution.^[45–47]^ Numerous cross-sectional studies echo our findings. An investigation in rural Sichuan, China, among older adults, revealed that physical activity significantly influences the orientation and memory dimensions of cognitive function.^[48]^ Additionally, a study encompassing 31,461 participants in India highlighted the positive impact of physical activity on cognitive function.^[49]^ Further, a survey involving 10,097 elderly individuals in Korea’s KLoSA found that physical activity effectively slows cognitive decline.^[50]^

The relationship between cardiovascular disease biomarkers and cognitive functions has garnered widespread attention. Cooper et al., observed a notable finding that aortic stiffness is associated with memory.^[51]^ In a longitudinal study by Zheng and Xie, high-sensitivity C-reactive protein was found to be inversely associated with cognitive function.^[52]^ Ogoh has reported the relationship between vascular function and blood flow regulation on cognitive function.^[53]^ Furthermore, the number of brain neurons is related to neurodegenerative diseases and cognitive function.^[54]^ Cognitive function can be effectively affected through the effect of vascular function on nerve cells. These results show the possibility that active participation in physical activities might increase blood flow supply, thereby providing sufficient oxygen and nutrients to the brain^[55]^ and protecting neuronal cells by enhance antioxidant activity.^[56]^

Earlier studies have consistently reported a correlation between physical activity and pulse wave velocity. In a study by Metsämarttila et al, they showed an inverse relationship between physical activity and carotid-femoral pulse wave velocity.^[57]^ Park et al, discovered that a combination of exercises effectively reduced pulse wave velocity in obese elderly men.^[58]^ Similarly, Fantin et al, reported the effects of physical activity on arterial stiffness in older women, as determined by cfPWV and carotid-radial pulse wave velocity (crPWV).^[59]^ McEniery et al conducted a 20-year follow-up study involving 825 men from the Caerphilly Prospective Study, revealing that blood pressure and inflammation were pivotal predictors of aortic stiffness.^[60]^ The findings of Kozakova et al, indicate that physical activity can alter arterial function by improving intracellular redox balance and mitochondrial health and reducing levels of systemic inflammatory markers.^[61]^ Another study by Mora et al underscored that physical activity exerts a more significant impact on inflammation and blood pressure than on other risk factors.^[62]^ Consequently, engaging in physical activity may play a crucial role in reducing blood pressure, mitigating inflammation, and ultimately diminishing aortic stiffness, thereby influencing pulse wave velocity.^[58]^

In our study, we found that approximately 19.35% of the effect of physical activity on cognitive function was mediated by ePWV, suggesting a potential mechanistic pathway linking lifestyle factors, vascular aging, and cognitive health. This highlights the role of ePWV as a mediator in this association. Our findings align with and extend recent advances in the field. Large-scale observational studies and meta-analyses have underscored the central role of vascular aging in cognitive decline, with arterial stiffness, systemic inflammation, and metabolic dysregulation identified as major contributors.^[63,64]^ Interventional trials further demonstrate that multidomain lifestyle strategies (such as structured exercise combined with dietary modification) can improve vascular function and, in turn, enhance cognitive performance in older adults.^[65–67]^ Beyond lifestyle behaviors, recent evidence highlights that nutritional and endocrine pathways also contribute to both vascular and cognitive health. For example, dietary quality and specific nutrient profiles have been linked to reduced arterial stiffness and better cognitive outcomes,^[68,69]^ while endocrine factors such as insulin resistance and hormonal dysregulation have been shown to accelerate vascular aging and cognitive decline.^[70,71]^ At the cellular level, oxidative stress, low-grade neuroinflammation, and vascular endothelial dysfunction are increasingly recognized as shared mechanisms underlying both elevated ePWV and impaired cognition.^[72]^ By quantifying the mediation effect of ePWV, our study adds novel empirical evidence to this growing body of work, suggesting that vascular stiffness may serve as a mechanistic pathway through which physical activity confers cognitive protection. These results further support the rationale for multidomain preventive strategies (integrating exercise, nutrition, and endocrine health management) to mitigate vascular aging and preserve cognitive function in older adults.^[73,74]^

Strengths of this work include the use of nationally representative NHANES data, standardized blood pressure and cognitive assessments, and extensive covariate adjustment with sensitivity analyses, which enhance the robustness of the findings. Nevertheless, several limitations should be acknowledged. First, the cross-sectional design restricts causal inference, and the use of self-reported questionnaires for physical activity may have introduced recall bias, likely leading to underestimation of true activity levels. Second, although we adjusted for multiple covariates, residual confounding (e.g., dietary patterns, psychosocial stress) cannot be ruled out, and such bias would most likely attenuate rather than exaggerate the observed associations. Third, ePWV was estimated rather than directly measured, which may introduce imprecision; moreover, errors in mean blood pressure measurement could further influence ePWV estimation. Overall, while the robustness checks support the stability of our findings, these limitations suggest that the reported mediated proportion likely represents a conservative estimate and should be interpreted in light of these methodological considerations. Future longitudinal studies with directly measured pulse wave velocity, repeated blood pressure assessments, and objective physical activity measures are warranted to confirm and refine these observations.

## 5. Conclusions

In conclusion, our study highlights a significant positive association between physical activity and cognitive function among older adults, with arterial stiffness as a mediating factor in this relationship. Notably, the significance of this study lies in its utilization of ePWV that is more practical than cfPWV for assessing arterial stiffness.

## Acknowledgments

We would like to thank participants of this study and the National Health and Nutrition Examination Survey for providing publicly available data. Thanks to Zhang Jing (Shanghai Tongren Hospital) for his work on the NHANES database.

## Author contributions

**Conceptualization:** Yaxiong Zheng, Soyoon Lee.

**Data curation:** Yaxiong Zheng, Hwi-Yeol Yun, Jae-Hyun Lee.

**Formal analysis:** Yaxiong Zheng, Jae-Hyun Lee, Wooyeon Jo, Soyoon Lee.

**Funding acquisition:** Sang Ki Lee.

**Methodology:** Yaxiong Zheng, Jaeho Jin.

**Project administration:** Sang Ki Lee.

**Software:** Jaeho Jin.

**Supervision:** Hwi-Yeol Yun, Sang Ki Lee.

**Validation:** Yaxiong Zheng, Hwi-Yeol Yun, Wooyeon Jo.

**Visualization:** Yaxiong Zheng, Seyeon Jang.

**Writing – original draft:** Yaxiong Zheng, Jae-Hyun Lee, Sang Ki Lee.

**Writing – review & editing:** Yaxiong Zheng, Sang Ki Lee.

## Supplementary Material
