# Supplementary material for: Mediation role of estimated pulse wave velocity in the association between physical activity and cognitive function
Source: Medicine (Baltimore). 2026 Apr 3;105(14):e48085. doi: 10.1097/MD.0000000000048085 (PMC13052969; doi:10.1097/MD.0000000000048085)
Supplement: Supplementary file 1 [file medi-105-e48085-s001.docx]

**Supplementary Material**

**Supplementary Table 1. Sensitivity analysis of mediation effect of ePWV on the association between physical activity and cognitive function**

| **Model / Subset** | **Covariates** | **Total effect**  **(c)** | **a path**  **(X→M)** | **b path**  **(M→Y)** | **Indirect effect (a×b)** | **Boot SE** | **Z** | ***P*-value** | **95% Boot CI** | **Direct effect (c')** | **Proportion mediated (%)** |
| --- | --- | --- | --- | --- | --- | --- | --- | --- | --- | --- | --- |
| Main model | Age, sex, ethnicity, marital status, education, BMI, hypertension, CVD, smoking, alcohol use | 0.031 | -0.125 | -0.046 | 0.006 | 0.002 | 3.68 | < .0001 | 0.003–0.009 | 0.025 | 19.35 |
| Model A | Age, sex | 0.031 | -0.120 | -0.045 | 0.005 | 0.002 | 3.50 | < .0001 | 0.002–0.008 | 0.026 | 16.13 |
| Model B | Main covariates | 0.031 | -0.125 | -0.046 | 0.006 | 0.002 | 3.68 | < .0001 | 0.003–0.009 | 0.025 | 19.35 |
| Model C | Extended covariates | 0.031 | -0.128 | -0.047 | 0.006 | 0.002 | 3.80 | < .0001 | 0.003–0.010 | 0.025 | 19.35 |
| Exclude CVD | Main covariates without CVD | 0.030 | -0.123 | -0.045 | 0.006 | 0.002 | 3.60 | < .0001 | 0.003–0.009 | 0.024 | 20.00 |
| Trim ePWV extremes | Main covariates | 0.031 | -0.125 | -0.046 | 0.006 | 0.002 | 3.68 | < .0001 | 0.003–0.009 | 0.025 | 19.35 |
| Male subgroup | Main covariates | 0.030 | -0.124 | -0.045 | 0.006 | 0.002 | 3.65 | < .0001 | 0.003–0.009 | 0.024 | 20.00 |
| Female subgroup | Main covariates | 0.031 | -0.126 | -0.046 | 0.006 | 0.002 | 3.70 | < .0001 | 0.003–0.009 | 0.025 | 19.35 |
| Age 60–69 subgroup | Main covariates | 0.032 | -0.127 | -0.047 | 0.006 | 0.002 | 3.75 | < .0001 | 0.003–0.010 | 0.026 | 18.75 |
| Age 70–79 subgroup | Main covariates | 0.031 | -0.125 | -0.046 | 0.006 | 0.002 | 3.68 | < .0001 | 0.003–0.009 | 0.025 | 19.35 |
| Age ≥80 subgroup | Main covariates | 0.030 | -0.123 | -0.045 | 0.006 | 0.002 | 3.60 | < .0001 | 0.003–0.009 | 0.024 | 20.00 |

Note: Main covariates: Age, sex, ethnicity, marital status, education, BMI, hypertension, CVD, smoking status, and alcohol use. Model A includes age and sex only. Model C (Extended covariates) additionally adjusts for diabetes, cholesterol, and other comorbidities to assess robustness. The indirect effect (a × b) was displayed to three decimal places (0.006), whereas the exact multiplication yielded 0.00575. This value was then used to calculate the proportion mediated (0.006 ÷ 0.031 = 19.35%). Due to rounding, the mediated proportion may differ slightly from that obtained using the exact value (≈18.6%).

**Supplemental digital content legends**

Supplementary Table 1. Sensitivity analysis of mediation effect of ePWV on the association between physical activity and cognitive function.

This table summarizes results from multiple sensitivity analyses performed to evaluate the robustness of the mediation models. Analyses included alternative covariate adjustments (age and sex only; main covariates; and an extended model including diabetes, cholesterol, and other comorbidities), exclusion of participants with cardiovascular disease, trimming of extreme ePWV values (1st–99th percentile), and subgroup analyses stratified by sex and age. Across all models, indirect effects remained consistent (approximately 0.005–0.006), 95% bootstrap confidence intervals did not cross zero, and the proportion mediated ranged from 15% to 22%, indicating a stable partial mediation effect of ePWV.
